# Sitting Time and Body Mass Index, in a Portuguese Sample of Men: Results from the Azorean Physical Activity and Health Study (APAHS)

**DOI:** 10.3390/ijerph7041500

**Published:** 2010-03-31

**Authors:** Rute Santos, Luísa Soares-Miranda, Susana Vale, Carla Moreira, Ana I. Marques, Jorge Mota

**Affiliations:** Research Centre in Physical Activity, Health and Leisure, Faculty of Sports, University of Porto, Portugal; E-Mails: rutemarinasantos@hotmail.com (R.S.); luisasoaresmiranda@hotmail.com (L.S-M.); susanavale@hotmail.com (S.V.); carla_m_moreira@sapo.pt (C.M.); anavalente@netvisao.pt (A.I.M)

**Keywords:** sitting, body mass index, obesity, sedentary behavior

## Abstract

The aim of this study was to verify the relation between body mass index (BMI) and sitting time in a sample of 4,091 Azorean men. BMI was calculated from self-reported weight and height. Total physical activity (PA) time and total sitting time were assessed with the IPAQ (short version). Linear Regression analysis showed that total sitting time (hours/day) was positively associated with BMI (B = 0.078; p < 0.001) after adjustments for age, meal frequency, alcohol and tobacco consumptions, island of residence, education level and total PA time. Although the cross sectional design precludes us from establishing causality, our findings emphasize the importance of reducing sedentary behavior to decrease the risk of obesity.

## 1. Introduction

Obesity has become a significant epidemic in both developed and developing countries due to its association with increased morbidity and mortality [1]. Overweight and obesity have also reached epidemic proportions in Portugal [2,3]. The consequences of obesity include physical, psychological and social aspects, which impact one’s quality of life. Although genetics is a strong component of obesity [4], lifestyle and environmental changes typical of industrialized societies are more likely to explain the recent obesity epidemic [5]. Indeed, obesity occurs when energy intake exceeds energy expenditure over time.

Physical inactivity has been viewed as one of the most important risk factors for coronary heart disease and other chronic diseases [6,7]. For instance, physical activity (PA) levels, as well as sedentary behavior appear to play an important role in long-term weight regulation [8]. Thus, the need to increase PA is a public health priority [9]. Current public health campaigns to reduce obesity and type 2 diabetes have largely focused on increasing PA and/or exercise, but have paid little attention to the reduction of sedentary behaviors [10]. Moreover, some studies have shown that non-exercise activity thermogenesis (NEAT) plays a key role in differences of energy expenditure in special populations such as the obese [11]. In fact, altering one’s postural allocation from a seated to standing position or engaging in light ambulation has been shown to significantly increase energy expenditure [11]. Conversely, it has been shown that workers performing their job functions in their usual fashion (seated) might expend more than 200 extra calories daily using a walking workstation [12]. Therefore, sitting is strongly and inversely associated with caloric expenditure that is likely an important cause of the obesity epidemic. Indeed, in our daily contemporary life, sitting is a predominant behavior for many hours per day [13].

Several studies have shown positive associations between BMI and different measures of sedentary behavior, such as TV viewing [14–16] motorized transportation [17,18] occupational sitting time [19] or sedentary behaviors during leisure time [20,21]. However, these studies fail to address total sedentary behavior and/or adjust the analysis for total PA.

In this context, the purpose of this study was to examine cross sectional relationship between body mass index (BMI) and total sitting time (*i.e.*, sitting across all three main domains: transportation, occupational and leisure time), adjusting the analysis for total PA time in Azorean adults.

## 2. Methods

### 2.1. Study Design and Sampling

Data for the present study are derived from the Azorean Physical Activity and Health Study. The study methods are reported elsewhere [3,22]. Briefly, data were collected in 2004 by mailing questionnaires to the adult residents of all the Azorean Islands and municipalities, a Portuguese archipelago. For the present study, only men who were employed (n = 3,939) or studying (n = 152) full-time and for whom questionnaires contained complete information on the variables of interest (*i.e.*, weight, height, PA, sitting, education level, meal frequency, sleep duration, smoking status and alcohol consumption) were included (n = 4,091, corresponding to 95.8% of the total sample of men). The decision to exclude unemployed or retired men was based on a previous study with this sample that showed that these groups of men were less likely to achieve higher levels of PA [23] and showed significantly higher total sitting time compared with men employed or studying full time (data not shown).

### 2.2. Measures

BMI [weight (kg) / height (m)^2^] was calculated from self-reported weight and height.

PA and sitting were assessed with the International Physical Activity Questionnaire (short last week version) and data was handled according to the IPAQ scoring protocol [24].

Total PA time was computed by multiplying the reported minutes of moderate and vigorous PA by the number of PA days of each type of PA. Subjects were categorized according to the ACSM/AHA PA guidelines: insufficiently active (participants who reported fewer than 150 min/week of at least moderate-intensity PA or less than 20 min/week of vigorous-intensity PA) and sufficiently active (participants who reported 150 min/week or more of at least moderate-intensity PA or 20 min/week or more of vigorous-intensity) [7].

The time spent sitting in an ordinary week day was considered a proxy measure of sedentary behavior. Subjects were categorized as having low or high total sitting time based on the median value for total sitting time found in this sample (180 min/day) [24].

Participants were also categorized into the following four groups: low total sitting time/sufficient PA time; low total sitting time/insufficient PA time; high total sitting time/sufficient PA time and high total sitting time/Insufficient PA time.

Other variables included in this analysis were:

- Education level: four years of education; 5–9 years of education; 10–12 of years education and higher education.
- Smoking: non smokers, former smokers, occasional smokers and current smokers.
- Alcohol consumption: non drinkers, former-drinkers, occasional drinkers, regular drinkers and heavy drinkers.
- Sleep duration: number of sleeping hours per day.
- Meal frequency: daily meal frequency was assessed by the question: “How many meals per day do you consume?” The main meals represented meals that were conventionally served on a plate.

### 2.3. Statistical Analysis

Statistical analyses were performed using the Statistical Package SPSS 17.0.

Data are presented as mean ± standard deviation unless stated otherwise.

Analysis of the variance with Bonferroni post-hoc tests, was used to assess BMI differences between sitting/PA groups.

Linear Regression analysis was performed to assess unstandardized regression coefficients and standard errors predicting BMI. Variables entered in the models were significantly correlated with BMI (in the bivariated analysis—data not shown). Model 1 was adjusted for age, education level; smoking, alcohol consumption, sleep duration, meal frequency, island of residence; model 2 was further adjusted for total PA time.

Statistical significance was set at p < 0.05.

## 3. Results

Of the 4,091 men included in this study, 96.3% were employed full-time. Of those, 73.4% of men had a low level of education (≤9 school years) and 71% were manual workers.

Participants had an average BMI of 26.3 ± 3.7 kg/m^2^ and spent on an average 25.5 ± 17.5 % of their waking hours sitting (about 250 min/day). Descriptive characteristics are presented in Table 1.

As depicted in Table 2, men with high sitting time and insufficient PA presented a highest mean of BMI −26.8 ± 3.8 kg/m^2^ (p < 0.05 for all).

Linear regression analysis showed that after adjustments for potential confounders total sitting time was positively associated with BMI (B = 0.078; p < 0.001—model 2).

## 4. Discussion

### 

To our knowledge, this is the first study to examine cross-sectional associations between BMI and total sitting time in Azorean adults. Our results show that men with high sitting time and insufficient PA presented the highest mean BMI compared to other sitting/PA groups. Linear regression analysis showed that after adjustments for potential confounders, total sitting time was positively associated with BMI.

It has been suggested that sedentary behavior should be explicitly measured either for surveillance purposes or research studies instead of being defined by lack of PA [25,26]. In fact, defining a sedentary population based on either low levels or lack of PA might be inaccurate because sedentary and PA are two independent behaviors with different effects on health outcomes [27–29]. Pate *et al.* (2008) consider that sedentary behavior refers to activities that do not increase energy expenditure substantially above the resting level, such as sitting, lying down or viewing TV among others [26].

It has been shown by Sugiyama *et al.* (2008) that among Australian adults, sedentary behavior and moderate to vigorous PA can be independent from each other and coexist. In this study, the authors demonstrated that even those meeting public health guidelines for leisure time PA may be at risk for overweight and obesity if they spend a large amount of their leisure time in sedentary activities [30]. Indeed, our data clearly shows that men with high total sitting time and with sufficient PA time presented on average a higher BMI compared with those with low total sitting time and sufficient PA time (p < 0.05).

Our results also indicate that total sitting time was positively associated with BMI after adjustments for potential confounders (including total PA time)—B = 0.078 (0.021), p < 0.001, in a sample that on average men only spend about 25% of their waking time sitting. These findings are in line with those found by others. Indeed, several studies have shown positive associations between BMI and different measures of sedentary behavior, such as TV viewing [14–16] motorized transportation [17,18] occupational sitting time [19] or sedentary behaviors during leisure time [20,21]. However, these studies fail to address total sedentary behavior and/or adjust the analysis for total PA. Therefore, our data extends previous findings by showing a positive association between total sitting time (considered as a proxy measure of total sedentary behavior) and BMI after adjustments for total PA time and other potential confounders in a large sample of adults.

#### Strengths and Limitations

The limitations of this study include the cross-sectional design, which does not allow for causal inferences; self-report measures of weight, height, PA and sitting; dietary intake was not assessed. Nevertheless, our data shows that total sitting time was positively associated with BMI independent of total PA time and other potential confounders, in a large sample of men (although the effect size of the association is small).

## 5. Conclusions

In conclusion, our results show that total sitting time was positively associated with BMI after adjustments for potential confounders (including total PA time). Although the cross sectional design precludes us from establishing causality our findings emphasize the importance of reducing sedentary behavior to decrease the risk of obesity.

## Figures and Tables

**Table 1.** Descriptive characteristics of the participants (mean ± SD).

	Men (n = 4,091)
Age	38.7 ± 9.3
BMI	26.3 ± 3.7
Total PA time (mean min/day)	77.2 ± 51.2
Total Sitting Time (min/day)	250.3 ± 172.8
% of sitting waking hours	25.5 ± 17.5

BMI = Body Mass Index; PA = Physical Activity.

**Table 2.** Mean (±SD) body mass index (kg/m^2^) by combined categories of total sitting time and total physical activity time.

	Men (n = 4,091)
Low total sitting time/Sufficient PA time	26.1 ± 3.8 *
Low total sitting time/Insufficient PA time	26.2 ± 3.7 *
High total sitting time/Sufficient PA time	26.3 ± 3.7 *
High total sitting time/Insufficient PA time	26.8 ± 3.8

Differences between combined categories of total sitting time and total physical activity time assessed with ANOVA (Bonferroni post-hoc).

* p < 0.05 different from High total sitting time/Insufficient PA time; PA = Physical Activity.

**Table 3.** Linear regression analysis (Unstandardized Coefficients and Std. Error) predicting Body Mass Index.

	Men (n = 4,091) Body Mass Index	
	Model 1 a	Model 2 b
Total Sitting Time (hours/day)	0.085 (0.021) *	0.078 (0.021) *

* p < 0.001;

a Model 1: Adjusted for age, meal frequency, alcohol and tobacco consumptions, island of residence and education level;

b Model 2: Adjusted for age, meal frequency, alcohol and tobacco consumptions, island of residence, education level and total physical activity.
